# Information Bottleneck Analysis by a Conditional Mutual Information Bound

**DOI:** 10.3390/e23080974

**Published:** 2021-07-29

**Authors:** Taro Tezuka, Shizuma Namekawa

**Affiliations:** 1 Faculty of Library, Information and Media Science, University of Tsukuba, Tsukuba, Ibaraki 305-8577, Japan; 2 Graduate School of Library, Information and Media Studies, University of Tsukuba, Tsukuba, Ibaraki 305-8577, Japan; kirua515@gmail.com

**Keywords:** conditional mutual information, information bottleneck, deep learning

## Abstract

Task-nuisance decomposition describes why the information bottleneck loss I(z;x)−βI(z;y) is a suitable objective for supervised learning. The true category *y* is predicted for input *x* using latent variables *z*. When *n* is a nuisance independent from *y*, I(z;n) can be decreased by reducing I(z;x) since the latter upper bounds the former. We extend this framework by demonstrating that conditional mutual information I(z;x|y) provides an alternative upper bound for I(z;n). This bound is applicable even if *z* is not a sufficient representation of *x*, that is, I(z;y)≠I(x;y). We used mutual information neural estimation (MINE) to estimate I(z;x|y). Experiments demonstrated that I(z;x|y) is smaller than I(z;x) for layers closer to the input, matching the claim that the former is a tighter bound than the latter. Because of this difference, the information plane differs when I(z;x|y) is used instead of I(z;x).

## 1. Introduction

Mutual information is now widely used to investigate the process of machine learning [1,2,3,4,5,6]. One notable example is information bottleneck theory [7]; when *x* is the input, *y* is the desired output, and *z* is the latent variables, the theory proposes using mutual information I(z;x) and I(z;y) to analyze the dynamics of learning. The authors postulated that supervised learning aims to reduce the information bottleneck loss I(z;x)−βI(z;y).

Recently, Achille and Soatto provided a fundamental analysis of information bottleneck theory using task-nuisance decomposition [8]. They proved that the second term I(z;x) in the information bottleneck loss bounds mutual information I(z;n) between the hidden layer activity and the nuisance.

In this paper, we propose to use conditional mutual information as an alternative criterion for bounding I(z;n) and suggest its use in the analysis of neural networks by information bottleneck theory.

Note that variables *x*, *y*, *z*, and *n* can be vectors, but we do not represent them using a bold font since the difference between scalar and vector is irrelevant to our analysis.

## 2. Related Work

### 2.1. Information Bottleneck Theory

Information bottleneck theory provides a unified view towards understanding machine learning models that have latent variables [7,9,10,11,12]. According to the theory, supervised learning aims to minimize the loss objective L=I(z;x)−βI(z;y), where β is a parameter that determines preference over the tradeoff between two terms. Since the latent variable *z* usually has a dimension lower than that of the observed variable *x* (as in convolutional neural networks), reducing I(z;x) while maintaining I(z;y) implies that information about *y* contained in *x* is compressed into *z*.

An effective compression of *x* should keep most information about *y* but reduce information about *x*. A learning algorithm can realize that by reducing I(z;x) while maintaining I(z;y). A predictor having such a representation removes frivolous transformations present in *x* while keeping information regarding *y*. Note that *y* is the ground-truth class and is different from the output $\hat{y}$ of a predictor. After learning, $p(\hat{y}|x)$ will be similar to p(y|x).

**Figure 1:**
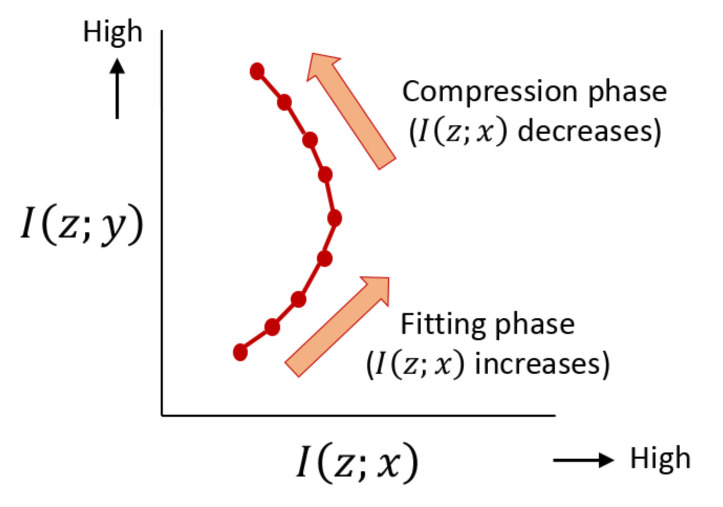
A schematic diagram of visualizing training dynamics using the information plane. Each dot represents a specific time point during the process of learning. In this example, the trajectory consists of two parts; fitting and compression phases. The fitting phase is where I(z;x) increases, and the compression phase is where I(z;x) decreases.

Information bottleneck theory has been applied to analyze the behavior of deep neural networks [13,14,15,16,17,18,19,20,21,22,23]. In this case, latent variables *z* correspond to hidden layer activities $z^{\langle \ell \rangle }$ for each layer *ℓ*. It has been suggested in [14] that the training process of deep learning may consists of fitting and compression phases, as represented in a schematic diagram in Figure 1. One possible use of our proposed bound is to conduct such an analysis in a more precise manner.

Fischer proposed a conditional entropy bottleneck defined by −H(z|x)+H(z|y)+γH(y|z), which is derived from I(z;x|y)−γI(z;y), where γ is a hyperparameter similar to β in an information bottleneck [24]. The use of conditional mutual information I(z;x|y) comes from the minimum necessary information (MNI) criterion, I(x;y)=I(x;z)=I(y;z). When this criterion is met, I(x;y|z)=I(x;z|y)=I(y;z|x)=0 is also true. In contrast, we derive the use of conditional mutual information by showing that I(z;x|y) forms an upper bound on I(z;n), where *n* is a nuisance variable. While Fischer claims that learning a compressed representation *Z* of *X* is equivalent to minimizing I(z;x|y), we show that reducing I(z;x|y) is even better than reducing I(z;x). We thereby provide solid ground to the conditional mutual information approach introduced by Fischer.

Geiger and Fischer introduced conditional mutual information I(z;x|y) by reformulating the information bottleneck functional I(z;x)−βI(z;y) to I(z;x|y)˘(β˘1)I(z;y)[25]. They defined a variational bound to the reformulated functional and analyzed its tightness. Our work sees I(z;x|y) from a different viewpoint, namely as a bound to I(z;n), where *n* is a nuisance variable.

Most recently, Yu et al. proposed deterministic information bottleneck (DIB) [26] based on matrix-based Rnyi’s α-order entropy functionals on positive definite matrices [27,28]. From these functionals, they defined Rnyi’s α-order mutual information $I_{\alpha }\left(A;B\right)$. Standard deep learning frameworks, such as PyTorch, can conduct automatic differentiation on $I_{\alpha }\left(A;B\right)$, enabling it to be trained using gradient descent. They also showed that the mutual information term acts as a regularization term.

### 2.2. Task-Nuisance Decomposition

Achille and Soatto [8] provided a new theoretical justification for information bottleneck theory. They introduced a nuisance variable representing stochastic fluctuations present in *x* that are unnecessary for conducting the classification task. For example, in image classification, a nuisance can represent a frivolous transformation such as rotation and translation. In terms of probability, *n* is a nuisance if it is independent from *y* and a Markov chain $\left(y,n\right)\to x\to z\to \hat{y}$ holds. The first part, (y,n)→x, is due to the generative process of *x*. The true category *y* and nuisance *n* together affects *x*. For example, in the CIFAR-10 image dataset, the distribution of intensity for each pixel is determined by image class *y* and sample-specific transformations. The latter part of the Markov chain, $x\to z\to \hat{y}$, comes from the predictor’s structure having latent variables *z*. In neural networks, *z* corresponds to a hidden layer. $\hat{y}$ is the output of the network, which is the predicted category for *x*.

It can be shown that, when *z* is a sufficient representation of *x*, that is, I(z;y)=I(x;y), then I(z;x) is an upper bound of I(z;n)[8]. Hence, reducing I(z;x) results in decreasing I(z;n). Because the effects from frivolous transformations are removed from *z*, the predictor generalizes better.

### 2.3. Non-Parametric Estimation of Mutual Information

One obstacle to putting information bottleneck theory into practice is the difficulty of estimating mutual information. When random variables are discrete or when distribution families are known, mutual information can be estimated straightforwardly. On the other hand, if the random variables’ distribution families are unknown, mutual information must be estimated non-parametrically. It is known to be a notoriously tricky task. Kraskov et al. have shown that *k*-nearest neighbor estimation works well when random variables are low-dimensional. However, the error increases as the dimension of the random variables becomes higher [29]. Kandasamy et al. used the Von Mises expansion and influence functionals to estimate entropy and mutual information [30].

Belghazi et al. recently proposed mutual information neural estimation (MINE), which uses a neural network to approximate a lower bound of mutual information [31]. Exploiting the fact that neural networks are a universal approximator of functions, the lower bound is obtained by:

(1) $$\hat{I}\left(x,z\right)=\underset{f\in F}{\sup}\left\{\frac{1}{n}\sum_{i=1}^{n}f\left(x^{\left(i\right)},z^{\left(i\right)}\right)-\log\frac{1}{n}\sum_{i=1}^{n}\exp\left(f\left(x^{\left(i\right)},{\overset{ˇ}{z}}^{\left(i\right)}\right)\right)\right\},$$

where F is a set of functions achievable by a neural network. Pairs $\left\{\left(x^{\left(i\right)},z^{\left(i\right)}\right)\right\}$ come from joint distribution p(x,z), while samples $\left\{{\overset{ˇ}{z}}^{\left(i\right)}\right\}$ come from marginal distribution p(z). It has been used for analyzing mutual information between layers of neural networks [32,33].

## 3. Method

We first describe the notations used in this section. We then describe the mathematical properties of our proposed use of conditional mutual information. Finally, we provide a way to estimate conditional mutual information.

### 3.1. Notations

Let *a*, *b*, *c* be scalars or vectors of random variables. We use a semicolon to separate random variables that are subject to computing mutual information, as in

(2) $$I\left(a;b\right)=E_{p(a,b)}\left[\log\frac{p(a,b)}{p(a)p(b)}\right].$$

A vector of random variables can be expressed explicitly by separating their components by a comma.

(3) $$\begin{array}{c} I\left(a;b,c\right)=E_{p(a,b,c)}\left[\log\frac{p(a,b,c)}{p(a)p(b,c)}\right]\\ I\left(a,b;c\right)=E_{p(a,b,c)}\left[\log\frac{p(a,b,c)}{p(a,b)p(c)}\right]. \end{array}$$

Conditioning both joint and product distributions defines conditional mutual information I(a;b|c).

(4) $$I\left(a;b|c\right)=E_{p(a,b,c)}\left[\log\frac{p(a,b|c)}{p(a|c)p(b|c)}\right].$$

In some articles, conditional mutual information is defined without integrating out *c*, as in

(5) $$\tilde{I}\left(a;b|c\right)=E_{p(a,b|c)}\left[\log\frac{p(a,b|c)}{p(a|c)p(b|c)}\right].$$

Our definition corresponds to taking the expectation of $\tilde{I}\left(a;b|c\right)$ by p(c), that is, $I\left(a;b|c\right)=E_{p(c)}\left[\tilde{I}\left(a;b|c\right)\right]$.

**Figure 2:**
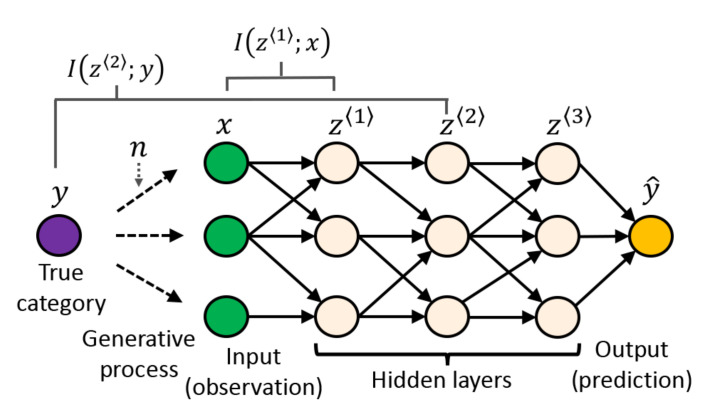
An example of a feed-forward neural network and the mutual information between layers. *y* is the true category of a sample. Observed signal *x* is generated from a distribution parametrized by *y* and contains fluctuations represented by a nuisance variable *n*. A neural network transforms the input to latent representations $z^{\langle \ell \rangle }$. The output of the network is $\hat{y}$, which is an estimate of *y*.

When applying our proposed framework to analyzing a neural network, *z* represents the hidden layer activities, *x* is the input, and *y* is a one-hot vector representing the ground-truth class label. In a feed-forward neural network, *z* can represent activities of any of the layers. When indicating the activity of layer *ℓ*, we use $z^{\langle \ell \rangle }$. Figure 2 illustrates an example of a feed-forward neural network.

A random variable *n* is a nuisance for *x* in performing task *y* if it affects *x* but is independent of *y*. For example, in image recognition, nuisances include translation, rotation, and small occlusions, which do not affect the object’s identity in the image. *z* is a representation of *x* if there is a (possibly non-deterministic) function that defines *z* by *x*. *z* is sufficient for the task *y* only if I(x;y)=I(z;y). It means that all information required to predict *y* present in *x* is also present in *z*.

### 3.2. Mathematical Property

To bound I(z;n), we propose using conditional mutual information I(z;x|y) instead of I(z;x), which is commonly used in information bottleneck theory. We prove that I(z;x|y) provides a tighter upper bound for I(z;n) than I(z;x). To do so, we use the following lemma, called the functional representation lemma, whose proof is given in [34]. It is also presented as Lemma C.1 in [8].

**Lemma** **1.** *Given a joint distribution p(x,y), where y is a discrete random variable, we can always find a random variable n independent of y such that x=f(y,n) for a deterministic function f.*

We now show that I(z;x|y) bounds I(z;n).

**Theorem** **1.** *Let n be a nuisance for the task y and let z be a representation of the input x. Suppose that z depends on y and n only through x. In other words, let random variables follow a Markov chain (y,n)→x→z. Then,*

(6) I(z;n)≤I(z;x|y).

**Proof.**  Let H(a|b) be either entropy or differential entropy, depending on the cardinality of the domain of *a*. Then,

(7) $$\begin{array}{ccc} I(z;n) & = & I(z;y,n)-I(z;y|n)\leq I(z;x)-I(z;y|n)\\ & = & I\left(z;x\right)-\left\{H(y|n)-H(y|z,n)\right\}\\ & \leq & I\left(z;x\right)-\left\{H(y)-H(y|z)\right\}\\ & = & H(z|y)-H(z|x)=H(z|y)-H(z|x,y)\\ & = & I(z;x|y). \end{array}$$

The first line is from the chain rule for mutual information (Theorem 2.5.2 in [35]). The first line is from the data processing inequality. The third line is because *y* is independent from *n*, and also because conditioning decreases entropy, that is, H(y|z,n)≤H(y|z). The fourth and the fifth lines are from the Markov chain. □

The theorem shows that conditional mutual information I(z;x|y) can bound I(z;n) even when *z* is not sufficient, in contrast to Achille and Soatto’s Proposition 3.1, which requires *z* to be sufficient [8]. It makes our theorem appealing since the sufficiency condition may not be fulfilled in general. Even in that case, our theorem makes task-nuisance decomposition applicable.

One question is, what is the difference between I(z;x), used in [8], and I(z;x|y), used by us. The following proposition answers this question.

**Proposition** **1.** *When random variables y, n, x, and z follow a Markov chain (y,n)→x→z, then*

(8) I(z;x)−I(z;x|y)=I(z;y).

*Furthermore, if z is sufficient, I(z;x)−I(z;x|y)=I(x;y).*

**Proof.**  (9) $$\begin{array}{ccc} I(z;x|y) & = & I(z;x,y)-I(z;y)\\ & = & I(z;x)+I(z;y|x)-I(z;y)\\ & = & I(z;x)-I(z;y). \end{array}$$

The first and second lines are from the chain rule for mutual information, and the third line is from the Markov chain. When *z* is sufficient, I(z;y)=I(x;y) by definition. □

The proposition shows that, instead of I(z;x|y), one can use I(z;x)−I(z;y) for bounding I(z;n). If *z* is sufficient, I(z;n) can also be bounded by I(z;x)−I(x;y). However, estimated mutual information often contains some errors. Estimating two values of mutual information may double that.

Let us note that lowering the upper bound does not necessarily reduce the objective function. However, in practice, upper bounds are commonly used as a surrogate objective. This may be because if a learning algorithm reduces an upper bound indefinitely, it will eventually reduce the objective function. Much of the existing work in machine learning relies on the assumption that reducing or raising bounds also reduces or raises the objective function, respectively.

Furthermore, many approximators in machine learning are formulated either as an upper or lower bound. Since I(z;x)−I(x;y) in [8] is the difference of two terms, neither an upper bound alone nor a lower bound alone can bound it. To bound I(z;x)−I(x;y), a combination of an upper bound and a lower bound is necessary. For example, if f(a,b) is an upper bound to I(a;b), f(z,x)−f(x,y) does not necessarily upper bound I(z;x)−I(x;y), due to the negation of I(x;y). By the same token, if g(a,b) is a lower bound to I(a;b), g(z,x)−g(x,y) does not necessarily lower bound I(z;x)−I(x;y). In contrast, I(z;x|y) does not contain a term with negation and avoids such a limitation.

There are many bounds on mutual information now and there will be more in the future. However, each bound has different strengths and weaknesses, such as asymptotic behavior, robustness and computational efficiency. If using two bounds, the resulting approximation will carry weaknesses from the two. It is often better to rely on only one approximator.

### 3.3. Estimation

Estimating mutual information for random variables with unknown distributions is a challenging task. It is even more so for high-dimensional random variables. Consequently, estimating conditional mutual information is also difficult. In this paper, we used MINE ([31]) to tackle this problem.

#### 3.3.1. Conditional MINE (CMINE)

To estimate conditional mutual information I(z;x|y), we group samples by class label *y*, compute an estimate by MINE for each group and take the weighted average of the estimates. In other words, we use

(10) $$\hat{I}\left(z;x|y\right)=\frac{1}{\sum_{c}m_{c}}\sum_{c}m_{c}\hat{I}\left(z;x|y=c\right),$$

where $\hat{I}\left(z;x|y=c\right)$ is the estimated value obtained by MINE using only samples in class *c* (i.e., y=c). $m_{c}$ is the number of samples in class *c*. We will call this estimation method conditional MINE (CMINE). Currently, the method can only be used when *y* takes discrete values.

CMINE estimates mutual information multiple times, but all in the form of I(z;x|y=c), where each term is not affected by the dimension of *y*. When the output variable *y* is high-dimensional, for example, in natural language processing, estimating I(x;y) likely results in a significant amount of error. Using I(z;x)−I(x;y) to compute I(z;x|y) is vulnerable to such errors, but CMINE can avoid such a limitation.

#### 3.3.2. Averaged MINE (AMINE)

In Section 5, we compare I(z;x|y) and I(z;x) using these estimates. We need to confirm that the number of samples used in estimation will not affect the comparison. When there are *m* samples and *h* possible values of *y* in I(z;x|y), CMINE applies MINE to roughly ⌊m/h⌋ samples for each possible value of *y*. Using fewer samples might lower the estimated mutual information since they may fail to capture the stochastic dependency between variables *x* and *z*. To avoid such unfairness, we used an estimator for I(z;x) that enforces the same restriction regarding the number of samples. Specifically, we randomly split the dataset into groups with the same sizes as grouping by class labels. We then run MINE for each group and compute the weighted average of the resulting estimates. We named this method averaged MINE (AMINE).

Specifically, let $c_{i}$ be the class label (i.e., the value of *y*) for the *i*-th sample. Define ρ as a random permutation of 1,…,n, where *n* is the number of samples in the dataset. We give a new label $c_{\rho (i)}$ to the *i*-th sample. In other words, we shuffle values of *y* across samples in the whole dataset. We then group samples following the new labels, compute MINE for each group, and average them using the number of samples in each group as weights.

## 4. Implementation

### 4.1. Dataset, Architecture, and Parameters

We used the MNIST, Fashion MNIST, and CIFAR-10 datasets for evaluation. Samples are images labeled by one of ten classes. Accordingly, *y* is a 10-dimensional one-hot vector. *x* is a vector obtained by flattening an image.

To observe mutual information between layers of a trained target neural network, we implemented a system that uses CMINE and AMINE. Table 1 indicates the architecture of the target network. One characteristic of the target network is that almost all layers have the same number of nodes. When the numbers of nodes are different between layers, the dimensions of $z^{\langle \ell \rangle }$ will differ, and it can affect the amount of error when estimating mutual information. Such variations would make a comparison between layers difficult.

The structure of MINE used in this paper is also shown in Table 1. Conv(a,b,c;d) is a convolution layer using a kernel of size a×b, with *c* channels and stride *d*. FC(*a*) is a fully-connected network with *a* nodes. We used ReLU as the activation function for each layer.

We implemented the networks using PyTorch, and trained them using an NVIDIA Quadro RTX 8000 with 48 GB memory. Table 2 shows the hyper-parameters used for optimizing the networks. After training, the target network achieved 96.3% test accuracy for classifying images in MNIST, 87.1% for Fashion MNIST, and 46.1% for CIFAR-10.

### 4.2. Preprocessing before Estimation by MINE

We used singular value decomposition (SVD) to reduce the dimension of the hidden layer activity *z*. It decreases computation time and also can reduce estimation error resulting from the high-dimensionality of the random variables. Since the task was classification into 10 classes, we chose 4, 8 and 12 as the reduced dimension. Without dimension reduction, the learning curves fluctuated rapidly and, upon observation, did not converge.

### 4.3. Cluttering

To observe the effect of a nuisance on the mutual information between layers, we conducted artificial occlusion experiments [8,36]. We generated cluttered images by superposing randomly allocated squares on top of images in the datasets. The squares can overlap. We used them as inputs to already-trained target neural networks. Then, we observed the activities of layers and estimated the mutual information between them. Each square has zero intensity on a randomly selected channel, and its size was 4×4 pixels. We tested by adding 64 squares to each image. They were added only when estimating mutual information and not during training of the target network.

## 5. Experiments

We conducted experiments to see how CMINE estimates conditional mutual information between layers in a neural network. In this section, $\hat{I}\left(a;b\right)$ and $\hat{I}\left(a;b|c\right)$ indicate estimates obtained by AMINE and CMINE, respectively, for mutual information I(a;b) and I(a;b|c). We used 10,000 samples to train the target network and 50,000 samples to estimate mutual information. When estimating mutual information, we recorded inputs *x*, desired outputs *y*, and hidden layer activities $z^{\langle \ell \rangle }$ from each layer *ℓ* of the target network.

### 5.1. Comparison of $\hat{I}\left(z^{\langle \ell \rangle };x\right)$ and $\hat{I}\left(z^{\langle \ell \rangle };x|y\right)$ across Layers

**Figure 3:**
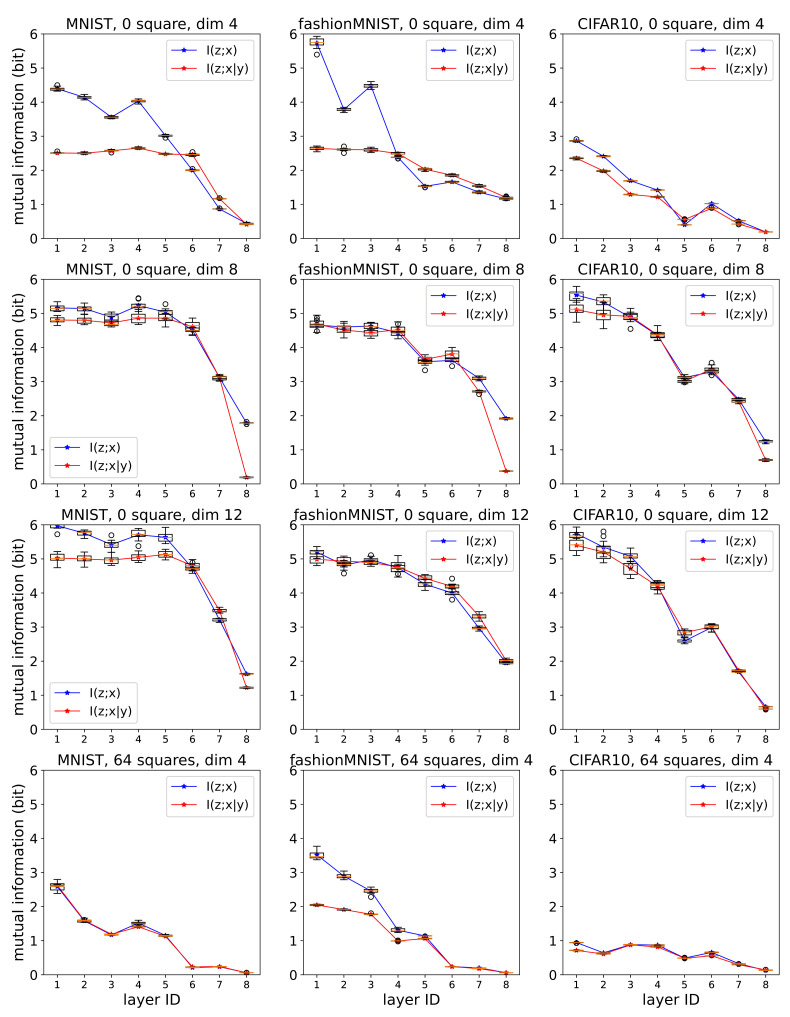
$\hat{I}\left(z^{\langle \ell \rangle };x\right)$ and $\hat{I}\left(z^{\langle \ell \rangle };x|y\right)$ obtained by AMINE and CMINE, respectively. *x* is the input, *y* is the output, and $z^{\langle \ell \rangle }$ is the activity of the *ℓ*-th layer. Horizontal axis represents different layers, with smaller numbers closer to the input. Vertical axis represents the value of estimated mutual information in bits. The boxes extend from the lower to upper quartile values for ten trials, with a line at the median. The whiskers extend from the boxes to show the ranges of the values across trials. The dimensions after SVD and the numbers of squares added for cluttering were compared.

Figure 3 shows a comparison of $\hat{I}\left(z^{\langle \ell \rangle };x\right)$ and $\hat{I}\left(z^{\langle \ell \rangle };x|y\right)$, obtained by AMINE and CMINE, respectively. A smaller *ℓ* (Layer ID) means the layer is closer to the input. The results using different datasets and the dimensions after SVD are compared. When the dimension increased, $\hat{I}\left(z^{\langle \ell \rangle };x\right)$ and $\hat{I}\left(z^{\langle \ell \rangle };x|y\right)$ both increased, indicating information loss due to SVD. When squares are added, estimated mutual information decreased both for $\hat{I}\left(z^{\langle \ell \rangle };x\right)$ and $\hat{I}\left(z^{\langle \ell \rangle };x|y\right)$.

The graphs show that, in general, both $\hat{I}\left(z^{\langle \ell \rangle };x\right)$ and $\hat{I}\left(z^{\langle \ell \rangle };x|y\right)$ decrease as they get farther away from the input. This is consistent with the data processing inequality. The graphs also indicate that, for layers closer to the input, $\hat{I}\left(z^{\langle \ell \rangle };x|y\right)$ is smaller than $\hat{I}\left(z^{\langle \ell \rangle };x\right)$, especially for MNIST and Fashion MNIST. For some layers closer to the output, the inequality did not hold. We assume this is due to SVD and MINE being unable to find stochastic dependency between layers due to how information is represented in these layers.

The results in which $\hat{I}\left(z^{\langle h\rangle };y\right)<\hat{I}\left(z^{\langle \ell \rangle };y\right)$ for h<ℓ contradict the data processing inequality. A possible cause is that it is easier for MINE to capture stochastic dependency with *y* from $z^{\langle \ell \rangle }$ that is transformed with more layers to output the estimate $\hat{y}$. For such transformed representations, the functional relationship between *y* and $z^{\langle \ell \rangle }$ is simpler, and MINE may more easily reach the supremum pursued during optimization [31]. It can also be from the difference in how much mutual information is preserved when preprocessed by SVD. If the functional relationship between *y* and $z^{\langle \ell \rangle }$ is highly non-linear, SVD fails to preserve that relationship.

### 5.2. Information Planes

Information planes are used in information bottleneck theory to visualize the dynamics of mutual information during training of the target network [14]. The dynamics are visualized as a trajectory on a plane whose axes are I(z;y) and I(z;x). Achille and Soatto pointed out that I(z;n), rather than I(z;x), is more fundamental [8]. From our analysis, I(z;x|y) is closer to I(z;n) than I(z;x). Therefore, we suggest using I(z;y) and I(z;x|y) as the axes of the information plane.

**Figure 4:**
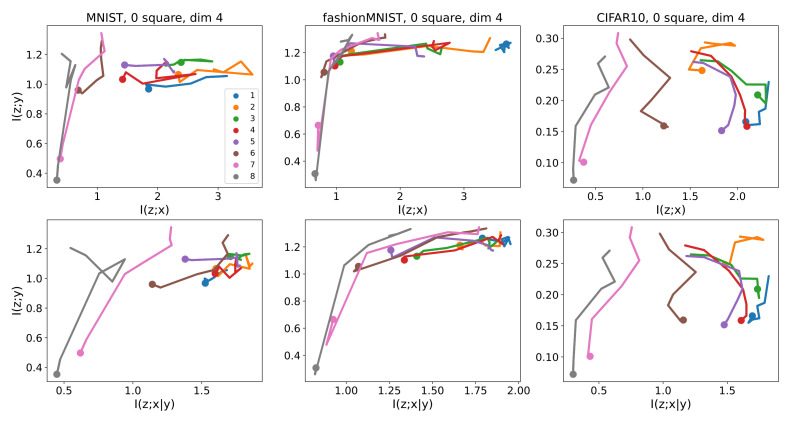
Information planes showing learning curves representing the dynamics of mutual information during training of the target neural network. The upper and lower rows are for $\hat{I}\left(z^{\langle \ell \rangle };x\right)$ and $\hat{I}\left(z^{\langle \ell \rangle };x|y\right)$, respectively. Each line corresponds to a layer of the target network. A line segment is placed every 10 batches, each batch containing 64 samples. Horizontal and vertical axes are mutual information in bits.

**Figure 5:**
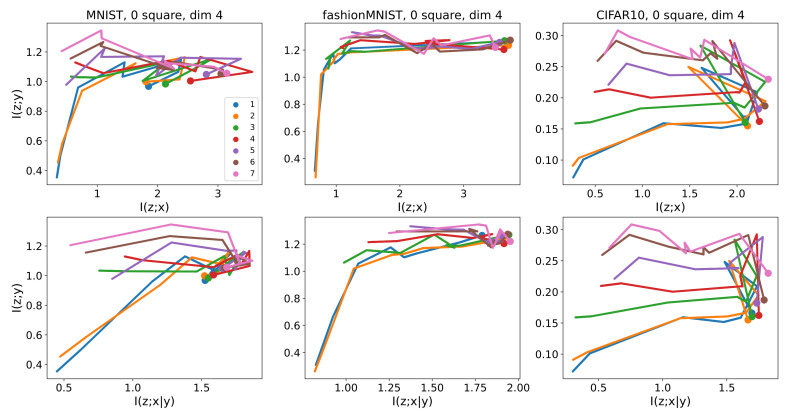
Information planes using the same conditions as Figure 4, but each line corresponds to a state of the whole target network (that is, for all layers) after training with a specific number of batches. The lines are drawn every 10 batches, each batch containing 64 samples. Horizontal and vertical axis are mutual information in bits.

To see the learning dynamics, we stopped training after every ten batches and estimated the mutual information. Each batch contains 64 samples. Figure 4 shows the resulting dynamics for images without cluttering squares. Each line represents a layer. On the other hand, in Figure 5, each line represents a batch. Note that the starting points are indicated by larger dots. The ranges of the horizontal axes are different between $\hat{I}\left(z^{\langle \ell \rangle };x|y\right)$ and $\hat{I}\left(z^{\langle \ell \rangle };x\right)$ since their values differ largely for some layers.

Figure 4 shows that some learning curves, for example, layers 5 to 8 for CIFAR-10, have the two-phased shape indicated in Figure 1. The shapes seem to be a little different between $\left(\hat{I}\left(z;x\right),\hat{I}\left(z;y\right)\right)$-coordinates and $\left(\hat{I}\left(z;x|y\right),\hat{I}\left(z;y\right)\right)$-coordinates for MNIST.

## 6. Conclusions

As a more precise way of conducting information bottleneck analysis, we proposed using conditional mutual information I(z;x|y) as an upper bound of I(z;n). We estimated values of conditional mutual information for a trained neural network using CMINE. The result showed that $I(z^{\langle \ell \rangle };x|y)$ could be used to observe information compression behavior of the neural network, similar to using $I(z^{\langle \ell \rangle };x)$ but with a tighter bound.

Our result suggests a new approach that uses I(z;x|y) instead of I(z;x) for information bottleneck theory. From Proposition 1, the information bottleneck loss $I\left(z;x|y\right)-\tilde{\beta }I\left(z;y\right)$ is equal to the original information bottleneck loss I(z;x)−βI(z;y) by setting $\tilde{\beta }=\beta -1$. However, the shapes of the trajectories in the (I(z;x|y),I(z;y))-coordinates would differ from those in the (I(z;x),I(z;y))-coordinates, and they can possibly provide more insights into the dynamics of compression and fitting in the process of learning.

The experiments showed some deviation from the data processing inequality. This is possibly due to the limitation of SVD and MINE in recovering stochastic dependency between layers. We believe more sophisticated dimension reduction and estimation methods may reduce errors. One approach would be to use a non-linear parametric dimension reduction method, such as a convolutional neural network (CNN), but it may require designing the network architecture appropriately. In addition to SVD, we also tried dimension reduction by CNN or global average pooling (GAP). Currently, however, the results are not as robust as those obtained by SVD.

Future work includes extending our scheme to tasks other than classification, for example, regression where *y* is a continuous variable. To do so, we must develop an estimation method of conditional mutual information I(z;x|y) other than CMINE. One possible way would be to combine CMINE with a nonparametric estimation method of p(y).

Since information bottleneck analysis by conditional mutual information is independent of how the mutual information is estimated, newly proposed estimators may improve the results. For example, the ensemble KDE-plugin estimator by Moon et al. [37] and the dependency graphs by Noshad et al. [38] could be used. Methods that directly estimate conditional mutual information, such as those by Singh and Póczos, are especially promising [39]. A variational bound to conditional mutual information proposed by Geiger and Fischer is another possible approach [25]. It is preferable to use an estimator that upper bounds mutual information since the purpose of using I(z;x|y) is to upper bound I(z;n). In the future, we expect there will be more methods that directly estimate conditional mutual information. Such a method will provide a further advantage to our formulation.

## Figures and Tables

**Table 1:** Architectures of the target network and MINE network; dim(a) is the dimension of the observed activity.

Target network	Conv(3,3,8;1) - Conv(3,3,8;1) - Conv(3,3,8;1) - Conv(3,3,8;1) - Conv(3,3,8;4) - FC(100) - FC(16) - Softmax(10)
MINE network	FC(dim(a)) - FC(100) - FC(100) - FC(100) - FC(1)

**Table 2:** Hyper-parameters used when training the target network.

	Optim.	Learn. Rate	# of Samples	Batch Size	Epochs
Target	Adam	0.001	10,000	64	100
MINE	Adam	0.001	50,000	32	30

## Data Availability

MNIST, Fashion MNIST, and CIFAR-10 datasets are available at http://yann.lecun.com/exdb/mnist/ (accessed on 29 January 2021), https://github.com/zalandoresearch/fashion-mnist (accessed on 29 January 2021), and https://www.cs.toronto.edu/~kriz/cifar.html (accessed on 29 January 2021), respectively.
